# 5-Year prognostic value of the right ventricular strain-area loop in patients with pulmonary hypertension

**DOI:** 10.1093/ehjci/jeaa143

**Published:** 2020-07-06

**Authors:** Hugo G Hulshof, Arie P van Dijk, Maria T E Hopman, Hidde Heesakkers, Keith P George, David L Oxborough, Dick H J Thijssen

**Affiliations:** 1 Department of Physiology, Research Institute for Health Sciences, Radboud University Medical Center, Philips van Leijdenlaan 15, 6525 EX Nijmegen, The Netherlands; 2 Department of Cardiology, Research Institute for Health Sciences, Radboud University Medical Center, Geert Grooteplein Zuid 10 6525 GA Nijmegen, The Netherlands; 3 Research Institute for Sport and Exercise Sciences, Liverpool John Moores University, Byrom Street, L3 3AF Liverpool, UK

**Keywords:** pulmonary hypertension, prognostic value, echocardiography, right ventricular function, ultrasound

## Abstract

**Aims:**

Patients with pre-capillary pulmonary hypertension (PH) show poor survival, often related to right ventricular (RV) dysfunction. In this study, we assessed the 5-year prognostic value of a novel echocardiographic measure that examines RV function through the temporal relation between RV strain (ϵ) and area (i.e. RV ϵ-area loop) for all-cause mortality in PH patients.

**Methods and results:**

Echocardiographic assessments were performed in 143 PH patients (confirmed by right heart catheterization). Transthoracic echocardiography was utilized to assess RV ϵ-area loop. Using receiver operating characteristic curve-derived cut-off values, we stratified patients in low- vs. high-risk groups for all-cause mortality. Kaplan–Meier survival curves and uni-/multivariable cox-regression models were used to assess RV ϵ-area loop’s prognostic value (independent of established predictors: age, sex, N-terminal pro B-type natriuretic peptide, 6-min walking distance). During follow-up 45 (31%) patients died, who demonstrated lower systolic slope, peak ϵ, and late diastolic slope (all *P* < 0.05) at baseline. Univariate cox-regression analyses identified early systolic slope, systolic slope, peak ϵ, early diastolic uncoupling, and early/late diastolic slope to predict all-cause mortality (all *P* < 0.05), whilst peak ϵ possessed independent prognostic value (*P* < 0.05). High RV loop-score (i.e. based on number of abnormal characteristics) showed poorer survival compared to low RV loop-score (Kaplan–Meier: *P* < 0.01). RV loop-score improved risk stratification in high-risk patients when added to established predictors.

**Conclusion:**

Our data demonstrate the potential for RV ϵ-area loops to independently predict all-cause mortality in patients with pre-capillary PH. The non-invasive nature and simplicity of measuring the RV ϵ-area loop, support the potential clinical relevance of (repeated) echocardiography assessment of PH patients.

## Introduction

Pulmonary hypertension (PH) is a progressive pulmonary vascular disease, which is associated with a poor 5-year survival rate.^1^ The primary cause of death relates to deterioration of right ventricular (RV) function, caused by the inability of the right ventricle to overcome the increased afterload.^2^ Approximately 44% of all deaths in patients with PH is caused by RV failure or sudden death.^3^ Despite the inherent connection between PH-related death and RV function, current risk assessment guidelines only includes cardiac index [derived by invasive right heart catheterization (RHC)] and right atrial (RA) area as variables of RV function.^4^ Given the invasiveness of RHC, associated risks/complications and inability for repeated measurements, alternative non-invasive measures of RV function may be more suitable in PH.

Although right heart echocardiography is advised in suspicion of PH and/or during follow-up of patients with PH, it possesses inferior prognostic value compared to other clinical measures [i.e. 6-min walking distance (6M-WD), N-terminal pro B-type natriuretic peptide (NT-proBNP)] and RHC.^4^ RV longitudinal ϵ (a relatively novel echocardiographic derived indices) possesses independent prognostic value for PH-related events and all-cause mortality^5^ and has been shown to be a stronger predictor than tricuspid annular plane excursion (TAPSE)^6^ in patients with pre-capillary PH.

Recently, we introduced the RV ϵ-area loop, which reflects the change of RV longitudinal ϵ across the cardiac cycle and is linked to the change in RV area.^7^^,^^8^ Simultaneous assessment of RV longitudinal ϵ and area provides novel insight into the contribution of RV longitudinal contraction and relaxation to area change. Interestingly, we found that the slope of the systolic ϵ-area relation is strongly related to pulmonary vascular resistance.^8^ This raises questions about the potential prognostic value of the RV ϵ-area loop for future PH-related events and (all-cause) mortality.

The primary aim of this study was to examine the prognostic value of characteristics of the RV ϵ-area loop for future all-cause mortality in patients with pre-capillary PH across a 5-year follow-up. We hypothesize that characteristics of the RV ϵ-area loop (e.g. slope of the systolic ϵ-area relation) possesses predictive value for all-cause mortality in patients with pre-capillary PH, independent from currently known predictors (i.e. age, sex, 6M-WD, NT-proBNP).

## Methods

### Ethics approval

Ethics approval was obtained from the Radboud University Medical Center ethics committee to perform the proposed work (reference number 2015-1832). This study was registered at the Netherlands Trial Register (NTR5230) and conforms to the standards set by the latest revision of the Declaration of Helsinki.

### Study population

We included 177 patients with pre-capillary PH, confirmed by RHC, who underwent transthoracic echocardiography at the Department of Cardiology of the Radboud University Medical Center (Nijmegen) between June 2003 and June 2017. Patients with multifactorial PH were included when pre-capillary PH was confirmed and PH-modifying therapy was prescribed. Due to inadequate 2D image quality for RV longitudinal ϵ analysis, 44 patients were excluded, resulting in a final cohort of 143 patients. Additional information regarding the included population can be found in *Table 1*.

**Table 1:** Population characteristics of the included patients with pre-capillary PH

	PH-patients (*n* = 143)			
Age (years)	61 ± 16			
Female (%)	100 (70%)			
Height (cm)	169 ± 9			
Weight (kg)	73 ± 15			
BSA (m^2^)	1.82 ± 0.19			
BMI (kg/m^2^)	26.0 ± 4.9			
Therapy at time of ultrasound				
Treatment naive	89 (62%)			
Single therapy	24 (17%)			
Double therapy	26 (18%)			
Triple therapy	4 (3%)			
Aetiology				
PAH	55 (38%)			
IPAH	40 (28%)			
CTEPH	26 (18%)			
Multifactorial	22 (15%)			
Risk factors	Yes	No	Unknown	Former
Hypertensive	41	39	63	
Dyslipidaemia	21	41	81	
Diabetes mellitus	15	52	76	
Smoker	16	41	27	59
Familiar history	34	41	68	

BMI, body mass index; BSA, body surface area; CTEPH, chronic thrombo-embolic pulmonary hypertension; IPAH, idiopathic pulmonary arterial hypertension; PAH, pulmonary arterial hypertension; PH, pulmonary hypertension.

### Experimental design

To address our aims, we retrospectively collected data on patient characteristics, PH-modifying therapy, 6M-WD and NT-proBNP at the time of echocardiographic assessment. Survival status of patients was retrieved from the Dutch population register at 21 January 2019, resulting in median follow-up of 60 (interquartile range 45–60) months while 91 patients fulfilled the maximal follow-up of 5 years.

### Echocardiographic assessment

Echocardiographic data were obtained by experienced sonographers using ultrasounds machines of the Vivid series (GE Healthcare, Horton, Norway). Data were stored in raw DICOM format in a password-protected archive of the Department of Cardiology of the Radboud University Medical Center. Data were retrieved for subsequent analysis by a single experienced researcher using commercially available software (EchoPac version 113.05, GE Healthcare, Horten, Norway). This researcher was blinded for the outcome during follow-up.

### Conventional echocardiographic assessment

Conventional echocardiographic indices were obtained in accordance with ASE Guidelines for echocardiographic assessment of the right heart.^9^ RV end-diastolic area (RVEDA) and RV end-systolic area (RVESA) were measured during the same cardiac cycle from a modified apical four-chamber orientation. RV fractional area change (RVFAC) was calculated as (RVEDA − RVESA)/RVEDA) * 100. TAPSE was determined using an M-Mode image for measuring the displacement of the tricuspid annulus.

### 2D myocardial speckle tracking

A modified apical four-chamber view, with a frame-rate of at least 40 frames per second, was used to assess simultaneous RV longitudinal ϵ and area. Images were optimized to ensure adequate endocardial delineation using gain, compression and reject. A region of interest (ROI) was drawn from the basal free to the basal septal wall enclosing the entire myocardium. Automatic analysis divided this ROI in six segments, the average of these segments (i.e. RV global longitudinal ϵ) was used in subsequent analysis.^7^ RV global longitudinal ϵ instead of RV free wall ϵ was used to ensure the inclusion of changes in RV function due to ventricular dyssynchrony as present in patients with pre-capillary PH.^10^

### RV ϵ-area loops

Temporal RV longitudinal ϵ values were exported to a spreadsheet (Excel, Microsoft Corp, Washington, US). To correct for differences in hazard ratio (HR) between subjects and length of the systolic and diastolic part of cardiac cycle, the temporal RV longitudinal ϵ values were divided in 300 points for systole and 300 points for diastole by cubic spline interpolation. For both systole and diastole, the 300 ϵ values were then split into 5% increments of the cardiac cycle providing 10 points in systole and 10 points in diastole. Concomitant time points, derived by tracing the echocardiography derived electrocardiogram signal, of the ɛ values were used in the same image and cardiac cycle to trace RV monoplane areas. For each patient, an RV ɛ-area loop was created.

**Figure 1:**
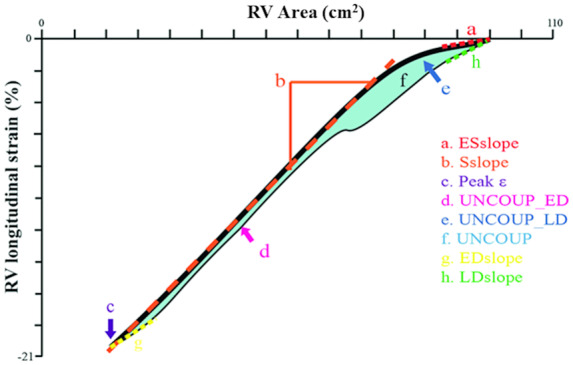
Schematic overview of the RV ϵ-area loop and the derived characteristics. The black line represents the ε-area loop, the thick part represents the systolic phase and the thin line the diastolic phase.

The RV ɛ-area loops were assessed by (i) the early systolic ɛ-area relation (ESslope), (ii) linear slope of ϵ-area relation during systole (Sslope), (iii) end-systolic peak ϵ (peak ϵ), (iv) diastolic uncoupling (i.e. mean difference between systolic vs. diastolic ϵ contribution to area change) during early filling (UNCOUP_ED), (v) diastolic uncoupling during late diastole (UNCOUP_LD), (vi) diastolic uncoupling during the entire cardiac cycle (UNCOUP), (vii) the early diastolic ɛ-area relation (EDslope), and (viii) the late diastolic ɛ-area relation (LDslope) as presented in *Figure 1*. Based on our extensive pilot work,^7^^,^^8^^,^^11^ we adopted either a linear regression (i.e. Sslope) or a second order polynomial (i.e. ESslope, UNCOUP_ED, UNCOUP_LD UCOUP, EDslope and LDslope) approach for data analysis as these models provide the best fit. Specifically, ESslope was calculated as the contribution of RV longitudinal ϵ to the first 5% of area change. The Sslope was derived as the gradient over the systolic phase of the RV ϵ-area loop. Longitudinal peak ϵ was derived as the raw peak ϵ value from the RV global longitudinal ɛ data. UNCOUP_ED, UNCOUP_LD, and UNCOUP were calculated as an normalized estimation of the area between the systolic and diastolic strain-area curves. For this purpose, systolic and diastolic ϵ values were calculated at each % increment of end-diastolic area (EDA). Subsequently, the difference between diastolic and systolic ϵ at each % of EDA was calculated. Based on individual RVFAC the working range of the ventricle was determined, after which UNCOUP_ED, UNCOUP_LD and UNCOUP were calculated as the mean of the differences at the lowest two-thirds of EDA’s, at the highest one-third of EDA’s and over the entire working range respectively. EDslope and LDslope were calculated as the contribution of RV longitudinal ϵ to the first and last 5% of area change respectively. In addition, we calculated the intraclass correlation (ICC) for intra-rater variability for all loop characteristics in a healthy population (*n* = 7), with exception of UNCOUP_LD, we retrieved good to excellent ICC (Supplementary data online, *Table S1*).

### Statistical analysis

Continuous variables were expressed as mean ± standard deviation in case of normal distribution. Normality of data distribution was examined using a Kolmogorov–Smirnov test. In case of non-Gaussian distribution, log-transformation was applied and data were presented as median (interquartile range). Categorical variables were expressed as percentage. Patients lost to follow-up were censored at the time of last available follow-up.

#### Cut-off values for risk stratification

Based on the optimal combination of sensitivity and specificity, derived from receiver operating characteristic curve (ROC) analyses at 5-year follow-up, cut-off values for all echocardiographic derived parameters were obtained (Supplementary data online, *Table S2*). Based on this cut-off value, patients were divided into low vs. high risk for all-cause mortality. Cut-off values for established predictors (6M-WD, NT-proBNP, and RA area) for low vs. high-risk group were based on current guidelines.^4^

#### Survival analysis

Kaplan–Meier survival curves were constructed to assess discriminative capacity of the RV ϵ-area loop characteristics. Univariate cox proportional HRs were determined to assess the predictive value of RV ϵ-area loop characteristics for all-cause mortality. Subsequently, significant univariate predictors were fitted into multivariable models to determine their independent predictive value compared to the reference model (consisting of age, sex, 6M-WD, and NT-proBNP). Finally, we calculated a combined RV loop-score based on the RV ϵ-area loop characteristics with predictive value after univariate cox regression analyses (*n* = 6, *Table 3*), combining the risk stratifications of the individual characteristics. The RV loop-score was ranged between 0 and 6 (i.e. 1 point for each characteristic in the high-risk category), categorizing patients with ‘low score’ (RV loop-score of 0–3) vs. ‘high score’ (RV loop-score of 4–6). First, we examined the Kaplan–Meier curve based on the RV loop-score. Secondly, we examined if the RV loop-score improved risk stratification based on the 2015 European Society of Cardiology/European Respiratory Society (ESC/ERS) guidelines for diagnosis and treatment of PH (including NT-proBNP, RA area, and 6M-WD) that is clinically used to categorize PH patients into low, intermediate and high risk.

## Results

Of the 143 patients, 117 were diagnosed with WHO class 1 PH, consisting of 95 patients with (idiopathic) pulmonary artery hypertension and 22 with multifactorial PH. The remaining 26 patients were diagnosed with WHO class IV PH, i.e. chronic trombo-embolic PH.

### Follow-up

**Figure 2:**
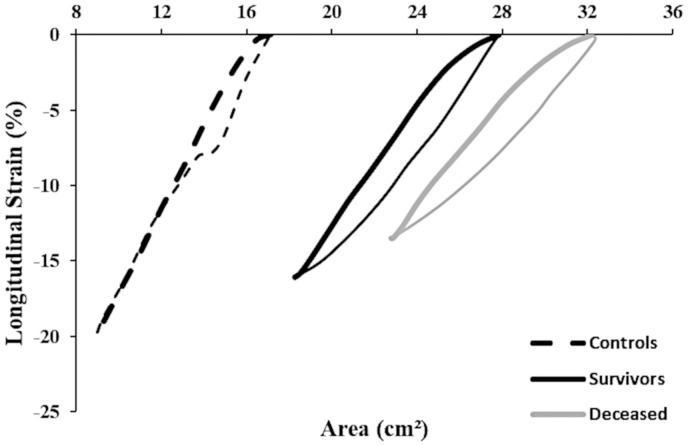
Mean RV ϵ-area loops taken at baseline (i.e. start of the follow-up period) from surviving patients (black ϵ-area loop, *n* = 98) and deceased patients (grey ϵ-area loop, *n* = 45). The dotted black lines represent the ϵ-area loop in a control group as published previously.^8^ The thick lines represents the systolic phase while the thin lines represent the diastolic phase of the ϵ-area loop.

**Figure 3:**
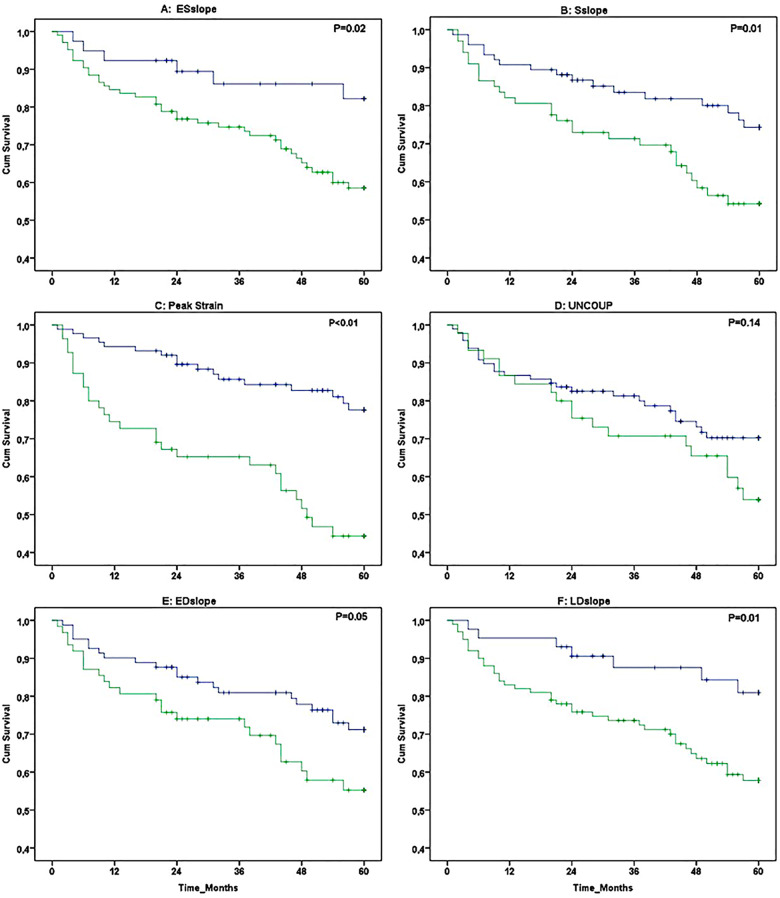
Kaplan–Meier survival curves (5-year follow-up) in 143 PH patients for individual characteristics of the RV ϵ-area loop that were categorized into low risk (blue line) and high risk (green line). The following loop characteristics were presented: ESslope (*A*), Sslope (*B*), peak strain (*C*), Uncoup (*D*), EDslope (*E*), and LDslope (*F*).

After a median follow-up period of 60 (45–60) months, 45 out of 143 patients died (5-year survival: 69%). Patients who died were older, predominantly male sex, had a higher NT-proBNP level, showed larger RVEDA and RVESA, and lower 6M-WD and RVFAC at baseline (all *P* < 0.05, *Table 2*). A marked rightward shift in the RV ϵ-area loop was visible at baseline between surviving and deceased patients (*Figure 2*). A significantly lower Sslope, EDslope, and peak ϵ was found in deceased vs. surviving patients after 5-year follow-up (all *P* < 0.05, *Table 2*). Kaplan–Meier survival analysis revealed significant differences in survival when patients were categorized based on ESslope, Sslope, Peak ϵ, EDslope, and LDslope of the RV ϵ-area loop (*Figure 3*).

**Table 2:** Population characteristics of the surviving and deceased patients after 5 years of follow-up

	60 (45–60) months of follow-up		
	Alive (*n* = 98)	Deceased (*n* = 45)	*P*-value
Demographics			
Age (years)	59 ± 17	64 ± 14	0.08
Height (m)	167 ± 0.09	169 ± 0.09	0.27
Weight (kg)	73 ± 15	74 ± 14	0.73
BSA (m^2^)	1.81 ± 0.19	1.84 ± 0.18	0.43
BMI (kg/m^2^)	26.0 ± 4.8	25.8 ± 5.0	0.86
Clinical characteristics			
6M-WD (m)	382 ± 112	290 ± 108	<0.01
Log NT-ProBNP	2.83 (1.07)	3.44 (0.95)	<0.01
Right heart catherization			
PAP (mmHg)	46 ± 15	43 ± 12	0.24
PVR (dynes*s/cm^5^)	652 ± 493	658 ± 329	0.95
CO (L/min)	4.8 ± 1.3	4.7 ± 1.9	0.84
CI (L/min/m^2^)	2.7 ± 0.8	2.5 ± 1.0	0.37
Echocardiography			
RVEDA (cm^2^)	28 ± 7	32 ± 9	<0.01
RVESA (cm^2^)	18 ± 6	23 ± 8	<0.01
RVFAC (%)	35 ± 7	31 ± 10	<0.01
TAPSE (cm)	2.0 ± 0.4	1.8 ± 0.4	0.06
RA area (cm^2^)	21 ± 7	22 ± 6	0.29
ϵ-area loop			
ESslope	−1.3 ± 1.0	−1.1 ± 1.0	0.23
Sslope (%/cm^2^)	−1.9 ± 0.8	−1.5 ± 0.6	<0.01
Peak ɛ (%)	−16.3 ± 4.5	−14.0 ± 4.7	<0.01
UNCOUP_ED (AU)	2.0 ± 2.4	1.7 ± 2.1	0.47
UNCOUP_LD (AU)	2.0 ± 2.4	1.8 ± 2.1	0.68
UNCOUP (AU)	2.0 ± 2.3	1.7 ± 2.0	0.52
EDslope (%/cm^2^)	1.3 ± 1.1	1.0 ± 0.8	0.25
LDslope (%/cm^2^)	2.2 ± 1.2	1.8 ± 0.9	0.02

6M-WD, 6 min walking distance; BMI, body mass index; BSA, body surface area; CI, cardiac index; CO, cardiac output; PAP, pulmonary arterial pressure; PVR, pulmonary vascular resistance; RVEDA, right ventricular end-diastolic area; RVESA, right ventricular end-systolic area; RVFAC, right ventricular fractional area change; TAPSE, tricuspid annular plane systolic excursion.

**Table 3:** Univariate Cox-regression hazard ratio’s of currently used predictors and echocardiographic derives indices of RV structure and function including the RV ɛ-area loop characteristics

	Univariate HR (95% CI)	*P*-value
Age (years)	1.023 (1.002–1.044)	0.03
Sex (male)	2.191 (1.210–3.968)	0.01
NT-ProBNP (>1400 ng/L)	3.215 (1.727–5.982)	<0.01
6M-WD (<165 m)	2.873 (1.005–8.209)	<0.01
RA area (>26 cm^2^)	1.310 (0.676–2.537)	0.42
RVEDA (>26.8 cm^2^)	2.777 (1.405–5.488)	<0.01
RVESA (>16.9 cm^2^)	3.690 (1.775–7.669)	<0.01
RVFAC (<25.5 %)	5.429 (2.973–9.914)	<0.01
TAPSE (<1.95 cm)	2.199 (1.202–4.022)	0.01
ESslope (>−1.695 %/cm)	2.658 (1.125–6.282)	0.03
Sslope (>−1.62 %/cm)	2.124 (1.161–3.886)	0.01
Peak ɛ (>−14.45 %)	3.400 (1.858–6.222)	<0.01
UNCOUP_ED (<1.025)	1.840 (1.025–3.301)	0.04
UNCOUP_LD (<2.035)	1.362 (0.745–2.491)	0.32
UNCOUP (<0.805)	1.557 (0.861–2.813)	0.14
EDslope (<0.95 %/cm)	1.800 (1.000–3.238)	0.05
LDslope (<2.465 %/cm)	2.684 (1.198–6.014)	0.02

6M-WD, 6 min walking distance; CI, confidence interval; HR, hazard ratio; RVEDA, right ventricular end-diastolic area; RVESA, right ventricular end-systolic area; RVFAC, right ventricular fractional area change; TAPSE, tricuspid annular plane systolic excursion.

### Uni- and multivariate Cox regression

Univariate cox regression analysis revealed age, sex, NT-proBNP, 6M-WD, RVEDA, RVESA, RVFAC, TAPSE, and RV ϵ-area loop characteristics (ESslope, Sslope, peak ϵ, Uncoup_ED, ESslope, and LDslope) as univariate predictors for 5-year all-cause mortality (*Table 3*). Multivariable models revealed that RVESA (>16.9 cm^2^), RVFAC (<25.55%), and peak ϵ (>−14.45%) remained significant predictors when added to the reference model (*Table 4*).

**Table 4:** Independent predictive value for 5-year survival of echocardiographic-derived parameters within a multivariable model, including, age, sex 6MWD, and log NT-proBNP as baseline model

	60 (45–60) months	
	45 events	
	HR (95% CI)	*P*-value
RVEDA (cm^2^)	1.566 (0.670–3.656)	0.30
RVESA (cm^2^)	2.520 (1.014–6.265)	0.05
RVFAC (%)	3.671 (1.635–8.238)	<0.01
TAPSE (cm)	1.322 (0.641–2.728)	0.45
ESslope (%/cm)	1.865 (0.707–4.924)	0.21
Sslope (%/cm)	1.089 (0.491–2.415)	0.84
Peak strain (%)	2.597 (1.135–5.943)	0.02
UNCOUP_ED (AU)	1.325 (0.662–2.653)	0.43
EDslope (%/cm)	1.347 (0.647–2.802)	0.43
LDslope (%/cm)	1.776 (0.711–4.435)	0.22

CI, confidence interval; HR, hazard ratio; RVEDA, right ventricular end-diastolic area; RVESA, right ventricular end-systolic area; RVFAC, right ventricular fractional area change; TAPSE, tricuspid annular plane systolic excursion.

### RV loop-score

**Figure 4:**
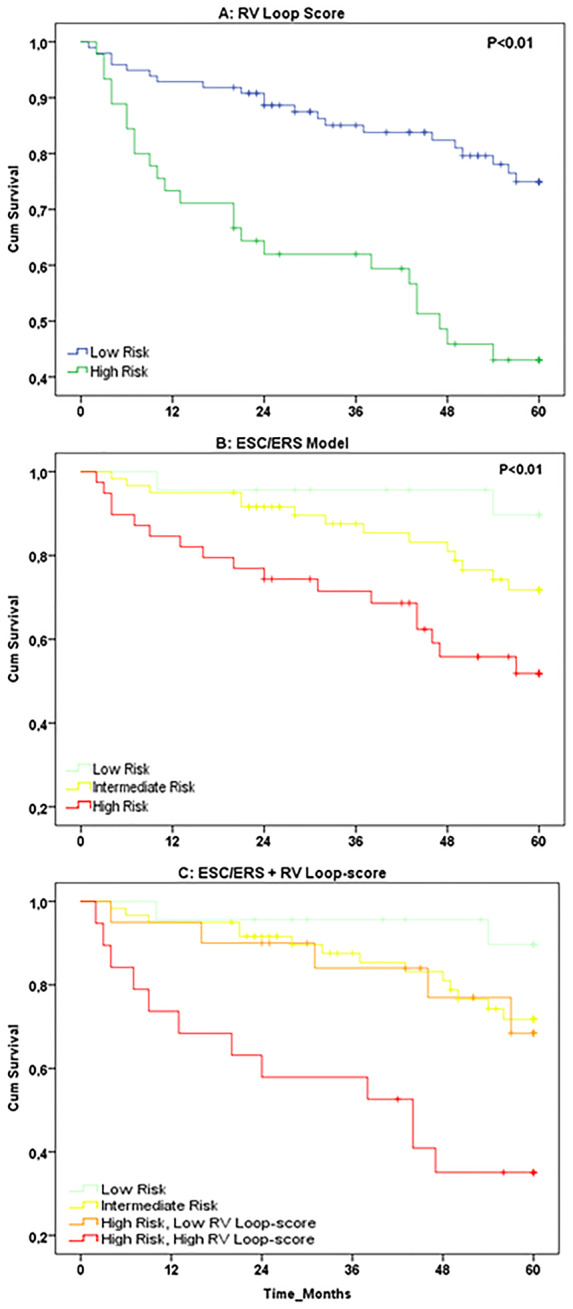
Kaplan–Meier survival curves for (*A*) the RV loop-score, categorised into low risk (blue line, *n* = 98) vs. high risk (green line, *n* = 45), (*B*) the 2015 ESC/ERS guidelines based model, categorised into low (green line, *n* = 23), intermediate (yellow line, *n* = 60), and high risk (red line, *n* = 39), and (*C*) the combined RV loop-score and ESC/ERS based model, categorized into low risk (green line, *n* = 23), intermediate risk (yellow line, *n* = 60), high risk—low RV loop-score (orange line, *n* = 20), and high risk—high RV loop-score (red line, *n* = 19).

Kaplan–Meier survival curves revealed significant differences in 5-year survival between ‘low’ and ‘high’ RV loop-scores (*Figure 4A*). HR showed a 3.182 (1.768–5.726) times higher risk for all-cause mortality in those with a ‘high’ RV loop-score compared to ‘low’ loop-score. More importantly, the RV loop-score improved risk classification following the 2015 ESC/ERS guidelines (*Figure 4B*), with high-risk individuals with ‘low’ RV loop-scores showing significantly better survival than high-risk patients with an ‘high’ RV loop-score (Kaplan–Meier: *P* = 0.02, *Figure 4C*). The RV loop-score did not significantly improve classification of patients at low (*P* = 0.83) and intermediate (*P* = 0.91) risk.

## Discussion

The purpose of this study was to examine the 5-year prognostic value of RV ϵ-area loop characteristics for all-cause mortality in patients with pre-capillary PH. We present the following findings: (i) A markedly different RV ϵ-area loop is present in PH patients who died across 5-year follow-up compared to surviving patients, (ii) RV ϵ-area loop characteristics show significant prognostic value for 5-year all-cause mortality in PH patients, with RV longitudinal peak ϵ possessing independent prognostic value, (iii) the RV loop-score, i.e. reflecting the number of ‘abnormal’ loop characteristics, successfully predicts 5-year all-cause mortality in PH patients but also improves risk stratification in the high-risk population. Taken together, our findings suggest the RV ϵ-area loop predicts all-cause mortality in patients with pre-capillary PH and may reclassify some patients from the high-risk group to an intermediate-risk group. The non-invasive nature and relative simplicity of measuring the RV ϵ-area loop, support the potential clinical relevance of echocardiography for (repeated) assessment of PH patients.

The marked shift between the RV ϵ-area loop of the surviving and deceased patients suggests the presence of a (further) impairment in RV function at the time of echocardiographic assessment in the deceased patients. The lower peak ϵ and flatter systolic ϵ-area slopes may be related to an impaired RV systolic function, presented by the smaller deformation (i.e. ϵ) of the ventricular wall for each cm^2^ change in area in the deceased patients compared to those who survived. These adaptations may be the consequence of the RV being exposed to increased afterload.^12^ However, no differences in mean pulmonary artery pressure or pulmonic vascular resistance were present between both groups. Possibly, different RV ϵ-area loop characteristics between groups may relate to the presence of maladaptation in the deceased group (i.e. dilation of ventricles).^13^ Similarly to the impaired systolic function, the lower diastolic ϵ-area slopes suggest that although RV area is increasing eventually, less contribution from longitudinal strain is present during early relaxation in deceased patients compared to those who survived. In line with our observation, others have shown increased isovolumetric relaxation times in patients with PH,^14^ indicating poor myocardial relaxation^15^ and diminished ventricular compliance. Taken together, both systolic and diastolic RV ϵ-area loop characteristics seem impaired in PH patients at higher risk for all-cause mortality across a 5-year follow-up.

Despite the growing consensus of the importance of RV function in patients with pre-capillary PH,^16^ current guidelines only include RA area, presence of pericardial effusion and through RHC obtained cardiac index to predict mortality.^4^ Interestingly, our study found no prognostic value of RA area, whilst measures the novel RV ϵ-area loop possessed predictive capacity. To further support the relevance of echocardiography, RVESA (>16.9), RVFAC (<25.5%), and RV longitudinal peak ϵ (>−14.45) possessed independent predictive value for all-cause mortality (*Table 4*). These results confirm findings of previous studies assessing the prognostic value of echocardiography in patients with pre-capillary PH.^17^^,^^18^ It is important to emphasize that we used ROC analyses to determine the threshold for low vs. high risk. A potential limitation of this approach is that these thresholds cannot be simply applied to other data sets. This highlights the importance of defining reference values for echocardiographic derived indices of RV function.

A key observation in our study was the prognostic value of both systolic and diastolic RV ϵ-area loop characteristics. Traditionally, markers of RV function only include RV systolic function. In a recent study, it was demonstrated that deterioration of RV diastolic function may precede deterioration of RV systolic function in patients with pre-capillary PH.^14^ This suggests that the processes of diastolic and systolic dysfunction represent linked, but possibly independent impact. In support of this view, we found only low-to-moderate correlations (*r*^2^ = 0.07–0.45) between indices of systolic function (ESslope, Sslope) and diastolic function (EDslope, LDslope) of the RV ϵ-area loop. This data highlights that the combined temporal data on the relative contribution of strain to area change during both systole and diastole, and the association between systolic and diastolic function, provide in depth insights in ventricular function compared to single peak value-based assessments such and peak strain or RVFAC. The dynamic temporal data acquired within the strain-area loop therefore increases its predictive value over functional measures at a single point during the cardiac cycle.

Presence of predictive value of the individual indices (including both systolic and diastolic RV function), and absence of strong relations amongst the six individual RV ϵ-area loop characteristics (*r*^2^ = 0.001–0.47), support the potential value of calculating a multi-parameter value such as an RV loop-score. Whilst the RV loop-score showed strong and significant prognostic value, adding the RV loop-score to the clinically used, 2015 ESC/ERS guidelines improved risk stratification for the high-risk population. More specifically, high-risk patients with a low RV loop-score showed a significantly better 5-year survival than those with a high RV loop-score. Effectively, the high-risk patients with low RV loop-scores were reclassified as moderate risk, given their similar survival curves (*Figure 4C*). This may be explained by the absence of echocardiographic RV function indices in the 2015 ESC/ERS risk stratification guidelines. Since deterioration of RV function remains the main cause of death in patients with pre-capillary PH,^16^ stratification of PH patients may be improved by including characteristics of RV function.

### Clinical implications

The prognostic capacity, but especially the ability of the RV ϵ-area loop to reclassify high-risk patients to intermediate-risk, has potential clinical importance. Following the 2015 ESC/ERS guidelines, predicting all-cause mortality and classifying PH patients importantly dictates clinical decision-making related to (non)pharmaceutical therapy. Specifically, excessive physical activity is not recommended in high-risk patients, whilst an increasing amount of follow-up visits and more aggressive PH-modifying therapy strategy is advised for high-risk patients. Successfully reclassifying the high-risk to intermediate-risk, i.e. 51% of our population, will therefore impact treatment (and lower associated costs and risks for complications/side-effects). Finally, the ability for repeated assessment of RV function enables evaluation of disease progress and efficacy of (non)pharmaceutical therapy.

### Limitations

Although all patients had pre-capillary PH, different aetiology was present. Whilst our sample size is sufficiently powered to identify predictors for all-cause mortality in PH, it does not allow for sub-analyses related to the various aetiology of PH. Another limitation is that some patients (*n* = 54) received PH-modifying therapy prior to inclusion. A sub-analysis revealed no differences in the RV ϵ-area loop characteristic at the time of inclusion between those with and without PH-modifying therapy prior to inclusion (Supplementary data online, *Table S3*). Moreover, patients with PH-modifying therapy at time of inclusion, typically started this within weeks prior to inclusion, whilst the majority started PH-modifying therapy within 1 week after the day of inclusion. Therefore, this short time-frame wherein all participants started PH-modifying therapy unlikely affected the main outcomes of our study. Finally, the current method to assess the ɛ-volume loops and their characteristics is currently only partially automated and thus time-consuming. Automated self-learning analysis protocols should be created prior to clinical implementation. In response to the time-consuming nature of the current loops analysis we have analysed a simplified parameter, here called the end-systolic-end-diastolic ϵ-area slope (ESEDslope), which provides the systolic slope based on individual measures of just RVEDA, RVESA, and Peak ɛ. Similar to the Sslope significant differences were found for ESEDslope between groups (Alive vs. Deceased; 1.80 ± 0.74 vs. 1.49 ± 0.55; *P* = 0.01) and a significant HR [2.084 (1.140–3.811); *P* = 0.02] using a univariate analysis. In line with the Sslope significance disappeared when ESEDslope was added to the reference model [HR 1.449 (0.663–3.168); *P* = 0.35]. This suggests that the combination of characteristics for the loop may outperform individual, simplified measures. This outcome supports the use of multiple measures from the ϵ-area loop in predictive analysis.

In conclusion, our data demonstrate a distinct RV ϵ-area loop in PH patients who deceased across a 5-year follow-up since diagnosis compared to those who survived. Several RV ϵ-area loop characteristics predict 5-year all-cause mortality, with RV peak longitudinal ϵ demonstrating independent prognostic value. More importantly, combining these RV ϵ-area loop characteristics into an RV loop-score successfully stratified PH patients into high vs. low risk for all-cause mortality, and improved risk stratification of the ‘high risk’ patients when added to the current (guidelines-based) risk assessment model. These results support the clinical potential of echocardiography-based assessment of the RV ϵ-area loop for risk stratification and survival-analyses in patients with pre-capillary PH. Future studies are warranted to further explore its potential use, especially in the context of repeated assessment of echocardiography to monitor progression and adjust treatment to optimize care for this vulnerable group of patients.

## Supplementary data

Supplementary data are available at *European Heart Journal - Cardiovascular Imaging* online.

## Data availability

The data underlying this article will be shared on reasonable request to the corresponding author.

## Funding

This study was supported by a junior researcher grant from the Radboud Institute for Health Sciences.

**Conflict of interest:** A.P.v.D. reports grants and personal fees from Actelion, outside the submitted work. All other authors reported nothing to disclose.

## Supplementary Material

jeaa143_Supplementary_Data
