# Supplementary material for: 5-Year prognostic value of the right ventricular strain-area loop in patients with pulmonary hypertension
Source: Eur Heart J Cardiovasc Imaging. 2020 Jul 6;22(2):188–95. doi: 10.1093/ehjci/jeaa143 (PMC7822639; doi:10.1093/ehjci/jeaa143)
Supplement: jeaa143_Supplementary_Data [file jeaa143_supplementary_data.zip › jeaa143-suppl_data/Supplementary table 1.docx]

**Supplementary table 1 –** Intra class correlation coefficients for intra-rater variability of the RV ԑ-area loop characteristics.

| N=7 | *Intra-Rater ICC* | *p-value* |
| --- | --- | --- |
| ESslope (%/cm) | 0.937 | **<0.01** |
| Sslope (%/cm) | 0.999 | **<0.01** |
| Peak ԑ (%) | 0.982 | **<0.01** |
| UNCOUP_ED (AU) | 0.777 | 0.06 |
| UNCOUP_LD (AU) | 0.371 | 0.32 |
| UNCOUP (AU) | 0.743 | 0.08 |
| EDslope (%/cm) | 0.665 | 0.09 |
| LDslope (%/cm) | 0.870 | **0.01** |
