# Supplementary material for: 5-Year prognostic value of the right ventricular strain-area loop in patients with pulmonary hypertension
Source: Eur Heart J Cardiovasc Imaging. 2020 Jul 6;22(2):188–95. doi: 10.1093/ehjci/jeaa143 (PMC7822639; doi:10.1093/ehjci/jeaa143)
Supplement: jeaa143_Supplementary_Data [file jeaa143_supplementary_data.zip › jeaa143-suppl_data/Supplementary table 2.docx]

**Supplementary table 2 –** The left column represents the by receiver operating curve (ROC) analyses derived Area’s under the curve of all echocardiographic derived indices. The middle and right column represent the derived cut-off values at the point of optimal sensitivity and specificity and their distribution over the low *versus* high risk categories.

|  | **AUC – 5-year** | **Cut-off** | |
| --- | --- | --- | --- |
|  |  | **Low-Risk** | **High Risk** |
| RVEDA | **0.649; p<0.01** | <26.8 | ≥26.8 |
| RVESA | **0.670; p<0.01** | <16.9 | ≥16.9 |
| RVFAC | **0.646; p<0.01** | >25.5 | ≤25.5 |
| TAPSE | **0.601; p=0.05** | >1.95 | ≤1.95 |
|  |  |  |  |
| ESslope | 0.579; p=0.13 | <-1.695 | ≥-1.695 |
| Sslope | **0.631; p=0.01** | <-1.62 | ≥-1.62 |
| PS | **0.647; p<0.01** | <-14.45 | ≥-14.45 |
| UNCOUP_ED | 0.551; p=0.33 | >1.025 | ≤1.025 |
| UNCOUP_LD | 0.522; p=0.68 | >2.035 | ≤2.035 |
| UNCOUP | 0.539; p=0.46 | >0.805 | ≤0.805 |
| EDslope | 0.543; p=0.41 | >0.95 | ≤0.95 |
| LDSlope | 0.598; p=0.06 | >2.465 | ≤2.465 |

RVEDA=Right ventricular end diastolic Area; RVESA=Right ventricular end systolic area; RVFAC=Right ventricular fractional area change; TAPSE=Tricuspid annular plane systolic excursion.
