# Supplementary material for: 5-Year prognostic value of the right ventricular strain-area loop in patients with pulmonary hypertension
Source: Eur Heart J Cardiovasc Imaging. 2020 Jul 6;22(2):188–95. doi: 10.1093/ehjci/jeaa143 (PMC7822639; doi:10.1093/ehjci/jeaa143)
Supplement: jeaa143_Supplementary_Data [file jeaa143_supplementary_data.zip › jeaa143-suppl_data/Supplementary table 3.docx]

**Supplementary table 3 –** Population based differences between the included patients who received PH-modifying therapy at time of inclusion and those who where treatment naïve.

|  | Treatment Naive  (n=89) | Therapy (n=54) | P-Value |
| --- | --- | --- | --- |
| ***Demographics*** |  |  |  |
| - Age (y) | 61±17 | 59±16 | 0.47 |
| - Length (m) | 1.67±0.09 | 1.70±0.10 | 0.06 |
| - Weight (kg) | 73±14 | 73±16 | 0.83 |
| - BSA | 1.81±0.18 | 1.83±0.22 | 0.61 |
| - BMI | 26.5±5.1 | 25.2±4.4 | 0.12 |
| - 6M-WD | 332±115 | 405±110 | **<0.01** |
| - NT-ProBNP | 2870±4636 | 2063±3315 | 0.29 |
|  |  |  |  |
| ***Echocardiography*** |  |  |  |
| - RVEDA (cm^2^) | 29±8 | 29±9 | 0.78 |
| - RVESA (cm^2^) | 20±7 | 19±8 | 0.76 |
| - RVFAC (%) | 33±8 | 34±8 | 0.55 |
| - TAPSE (cm) | 1.9±0.5 | 2.1±0.4 | **<0.01** |
|  |  |  |  |
| **ԑ-area loop** |  |  |  |
| - ԑ_ES (%) | -3.2±1.8 | -3.4±1.6 | 0.66 |
| - Sslope (%/cm^2) | -1.7±0.7 | -1.9±0.8 | 0.14 |
| - Peak ɛ (%) | -15.0±4.7 | -16.5±4.5 | 0.08 |
| - UNCOUP_ED | 1.8±2.2 | 2.0±2.5 | 0.69 |
| - UNCOUP_LD | 1.8±2.2 | 2.2±2.5 | 0.34 |
| - UNCOUP | 1.8±2.1 | 2.1±2.4 | 0.50 |
| - ESslope | 1.18±0.96 | 1.37±1.02 | 0.28 |
| - EDslope | 1.20±1.00 | 1.17±1.00 | 0.89 |
| - LDslope | 2.00±1.05 | 2.25±1.28 | 0.21 |

BSA=Body Surface Area; BMI=Body Mass Index; PAP=Pulmonary Arterial Pressure; 6M-WD=6 Minute Walking Distance; RVEDA=Right ventricular end diastolic Area; RVESA=Right ventricular end systolic area; RVFAC=Right ventricular fractional area change; TAPSE=Tricuspid annular plane systolic excursion.
